# Metal–Organic Frameworks Meet Metallic Oxide on Carbon Fiber: Synergistic Effect for Enhanced Photodegradation of Antibiotic Pollutant

**DOI:** 10.3390/ijms231911286

**Published:** 2022-09-25

**Authors:** Na Zhu, Sijie Zhou, Chunhua Zhang, Zhuan Fu, Junyao Gong, Zhaozixuan Zhou, Xiaofeng Wang, Pei Lyu, Li Li, Liangjun Xia

**Affiliations:** 1 State Key Laboratory of New Textile Materials and Advanced Processing Technologies, Wuhan Textile University, Wuhan 430200, China; 2 College of Textiles, Donghua University, Shanghai 201620, China; 3 Institute for Frontier Materials, Deakin University, Geelong, VIC 3216, Australia; 4 Institute of Textiles and Clothing, The Hong Kong Polytechnic University, Hong Kong 999077, China

**Keywords:** tetracycline hydrochloride, zinc oxide, zeolitic imidazolate framework–8, synergistic effect, water treatment, photocatalyst

## Abstract

Photodegradation shows a potential strategy for alleviating the excessive antibiotics crisis. The synergistic effect of various metal compounds immobilized on conductive substrates has been considered for wastewater treatment. However, developing a facile and universal approach for rational design and enhancing photocatalytic properties has endured extreme challenges. Herein, we develop a strategy to facilitate the photocatalytic reactions by designing a composite architecture of ZIF–8 ligand binding to the in–situ synthesis ZnO seed layer on carbon fiber. In this architecture, the dissolution and release of the seed layer in the excessive 2–Methylimidazole methanol solution were used as the binder to enhance the interplay between organic ligand and substrate. As an evaluated system for antibiotic contaminants, the photodegradation of tetracycline hydrochloride was performed with a removal efficiency of 88.47% (TC = 50 mg/L, pH = 4, 0.08 g of photocatalyst, illumination within 100 min). Moreover, the photocatalyst exhibited a steady photocatalytic activity (75.0%) after five cycles. The present work demonstrated a strategy for enhancing the photocatalytic performances of carbon fiber and accordingly provided useful perception into the design of the synergistic structure.

## 1. Introduction

With the development of the circular economy, the present wastewater treatment was designed with the philosophy of a low–carbon economy and environment–friendly to reduce energy consumption, emission of greenhouse gases, and waste generation [1,2]. Serious pollution of the water environment was assigned as the remarkable consequence of rapid global industrial development [3]. Pharmaceuticals have attracted wide attention due to their splendid competence in repairing for protecting health. Based on the broad spectrum and high activity, antibiotics were extensively applied for human, veterinary therapy and aquaculture, generating the wide distribution of the original molecule and degradation metabolites in various environments [4]. Meanwhile, as the extensively used medicine, the residual antibiotic has been discharged into the water environment, concealing a great threat to health and the balance of the aquatic environment [5,6], which have become urgently prominent. As one of the typical antibiotics, an effective method to reduce the content of intractable tetracycline hydrochloride (TC) in the water system is essential, excessive utilization of TC leads to the evolution of antibiotic resistance to bacterial reproduction, which has attracted considerable attention [7].

Owing to the chemical stability and solubility of TC, it is difficult to be removed from the water system through conventional waste treatment techniques such as filtration and precipitation. Therefore, the rapid and efficient transformation of TC to a non–toxic product is crucial for improving water quality and ecological stability. Advanced techniques including adsorption [8], photocatalysis [9,10], and ion exchange [11] were utilized to address pharmaceutical pollution problems. Among these, owing to the utilization of inexhaustible sunlight as the driving power, the attention of photocatalytic technology has been attracted as one of the most potentially advanced technologies to treat water, exhibiting clean, renewable, sustainable, and economic development. Semiconductor–based photocatalysts were regarded as a green potential material for photocatalytic degradation of TC in water [12,13]. Therefore, many efforts have been explored to enhance the photocatalytic activity for the removal of TC; Wang et al. [14] reported a WO_3_/g–C_3_N_4_ photocatalyst to degrade approximately 98.6% of TC, and Liu et al. [15] prepared a multiple–phase rGO/CuO/Cu_2_O heterostructures photocatalyst for boosting photocatalytic activity and durability of TC and displayed superior photocatalytic activity with a 99.8% degradation rate for TC. Moreover, Xu et al. [16] reported the in–situ growth of Ag_3_PO_4_ on calcined Zn–Al layered double hydroxides photocatalyst and the degradation efficiency of TC reached 96% within 90 min. Lin et al. [17] designed and synthesized CoS_2_/MoS_2_@Zeolite photocatalysts, which showed a superior photocatalytic efficiency of TC (96.71%) due to high active sites, separation, and transfer of electrons and holes in heterojunction. As a typical semiconductor with low cost and great conductivity, zinc oxide (ZnO) has played an irreplaceable role in the field of photocatalysis. In the ultraviolet (UV) region, ZnO reveals excellent light absorption performance with a broad bandgap of 3.37 eV [18]. As a crucial parameter, the adsorption performance has been focused on enhancing photocatalysis via combining various porous structures [19]. Optimization of the nanostructure combined with ZnO was considered an effective strategy [20]. The preferred porous materials of zeolitic imidazolate framework–8 (ZIF–8) were tacit, which consisted of unsaturated Zn^2+^ centers with 2–methylimidazole ligands [21,22], generating multiple extended 3D open frameworks. The unique structure provided properties of adsorption as well as an additional pathway for photoinduced electron migration, which promoted them and seem to be the potential candidates for photocatalysis with excellent chemical stability [23,24]. Based on these superior properties of ZIF–8, great efforts have been devoted to designing and synthesizing novel ZIF–8–based photocatalysts with excellent performance for removal TC, such as core–shell ZIF–8@In_2_O_3_ [25], core–shell g–C_3_N_4_@ZIF–8 [26], and MoS_2_/ZIF–8 hybrid materials [27], etc. However, the weak conductivity of ZIF–8 limited the charge transport [28]. Furthermore, more efforts were focused on the consideration of the substrate to enhance photocatalytic activity. As one of the most crucial photocatalytic material systems, carbon–based materials have been considered promising materials, due to their specific conductivity, stability, and changeable structures [29], such as the 3D nanofibrous structure of N–carbon@N–ZnO [30], hollow carbon fiber membrane with ZnO [31], 3D porous core–shell carbon fiber [32], Z–scheme carbon fibers@WO_3–x_ composites [33], and the carbon cloth of CoSe_2_@MoSe_2_ core–shell structure [34]. Combining different carbon–based materials with photocatalysts reduced charge recombination efficiency and enhanced the transmission of the photogenerated electron. More importantly, to solve the vexing problem of nanoparticle recycling, carbon–based materials with stable performance exhibited unique advantages, which could be easily recycled in abominable conditions.

Herein, the combination of ZIF–8 on the ZnO seed layer (ZZ–CF) was proposed to prepare a photocatalyst, which was further designed and fabricated immobilized onto a substrate of carbon fibers (CFs). In this hybrid structure, utilizing the dissolved and released ZnO formed at a low temperature in dimethylimidazole methanol solution to enhance the interplay between organic ligand and substrate. Meanwhile, carbon fibers substrate with faultlessly flexible, conductive, and stable properties exhibits the superior ability to facilitate electron transfer separation and reduce the recombination of photogenerated electron–hole pairs, which could significantly enhance photocatalytic performance. Furthermore, ZIF–8 on the seed layer in the 2–Methylimidazole methanol solution tune local aggregation promoting uniform coverage on CFs substrate. As an evaluated system, photodegradation of TC was performed with a removal efficiency of 88.47% and demonstrated excellent photocatalytic stability under ultraviolet–visible (UV–vis) light irradiation. This present work demonstrated a strategy for improving the photocatalytic performances of ZZ–CF and provided useful perception into the design of other photocatalysts for wastewater treatment, which shows potential for application in photocatalysis.

## 2. Results and Discussion

### 2.1. Structural Characterization

**Figure 1:**
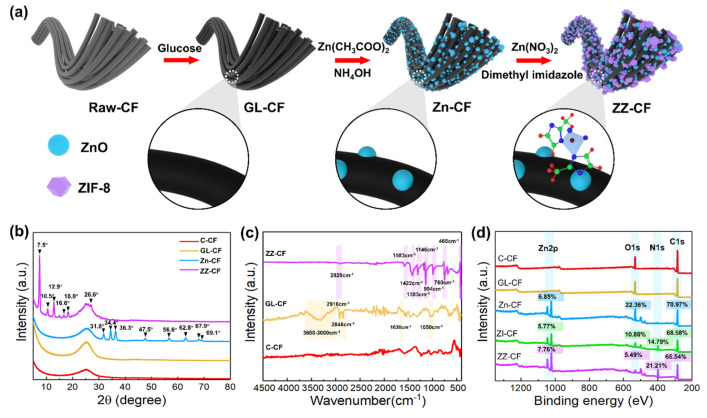
(**a**) Schematic diagram of the designed process for ZZ–CF photocatalysts; (**b**) XRD pattern of C–CF, GL–CF, Zn–CF, and ZZ–CF, respectively; (**c**) FTIR spectrum of R–CF, GL–CF, and ZZ–CF, respectively; (**d**) XPS spectra of C–CF, GL–CF, Zn–CF, ZI–CF, and ZZ–CF, respectively.

The strategy for reducing contact resistance and promoting photocatalytic activity has been developed to design the structure of ZZ–CF on conductive substrates. The superiority of the method was to decrease the tendency for the active photocatalytic layers to separate from the substrates, to enhance forces between the photocatalysts and substrates [32]. Meanwhile, a flexible photocatalyst has been designed successfully as an extensively applicable independent system. Therefore, attention has been attracted to the rationale design and synthesis of photocatalytic materials with unique architecture and morphology. Particularly, functional outer shell structures of ZIF–8 combined with the inner seed layer provide prominent structural advantages in photocatalyst applications. As shown in Figure 1a, the core–shell structure of ZZ–CF was obtained by hydrothermal reaction and a subsequent in situ synthesis three–step route. Glucose–activated carbon fiber was prepared using hydrothermal. Then, a thin layer of seed on the surface of the bath deposit in the Zn^2+^ solution was expected to provide an active site. Furthermore, utilization of the control ability of dimethylimidazole for Zn^2+^ release constructed a ZIF–8 shell structure, in which the ZnO seed layer exhibited a special function of dissolution–promoter and terrace–binder [20].

The corresponding XRD pattern confirms the existence of each component. XRD patterns of C–CF, GL–CF, Zn–CF, and ZZ–CF were shown in Figure 1b. The carbon coating on the surface of R–CF has not changed the crystalline structure of CF. The characteristic peaks located at 31.8°, 34.4°, 36.3°, 47.5°, 56.6°, 62.8°, 66.4°, 67.9°, and 69.1° were detected in the Zn–CF sample [28], which can be indexed to the (100), (002), (101), (102), (110), (103), (200), (112), and (201). Additionally, the characteristic XRD peaks, accordingly to the ZIF–8 crystal, located at 7.5°, 10.5°, 12.9°, 16.6°, 18.0°, and 26.6° were recorded in the ZZ–CF involved sample, which are ascribed to the (011), (002), (112), (022), (013), (222), and (134) [25]. However, unobvious characteristic peaks of the seed layer were recorded in the ZZ–CF sample which could be attributed to two aspects. On the one hand, the ZnO seed layer is dissolved and released Zn^2+^ ions to react with 2–methylimidazole and generate ZIF–8 truncated octahedrons on the ZnO surface, resulting in the ZnO loading content decreasing in the ZZ–CF sample further weaken the intensity of ZnO peaks in the ZZ–CF sample. On the other hand, it is worth noting that the ZnO is a core in the inner and the ZIF–8 a shell in the outer, resulting in the ultralow loading ZnO could be covered by ZIF–8 completely and showing in the SEM images, which causes the characteristic peaks for ZnO–CF could not be detected after doping ZIF–8. In this process, the excessive dimethylimidazole ligand demonstrated multiple roles for dissolution and terrace promoter on the seed layer [20]. The particular interactions contributed to a deterministic means of promoting substrate dissolution. XRD peaks of ZIF–8 were demonstrated in the ZZ–CF spectrum as the initial superfluous dimethylimidazole ligand, which was because etching ZnO was more toilless and self–assembled ZIF–8 sections formed when more Zn(NO_3_)_2_ solution was added [35]. This phenomenon indicated that the nucleation site, constituted by the template of metal oxide, can provide by sacrificing the metal ions released by itself.

**Figure 2:**
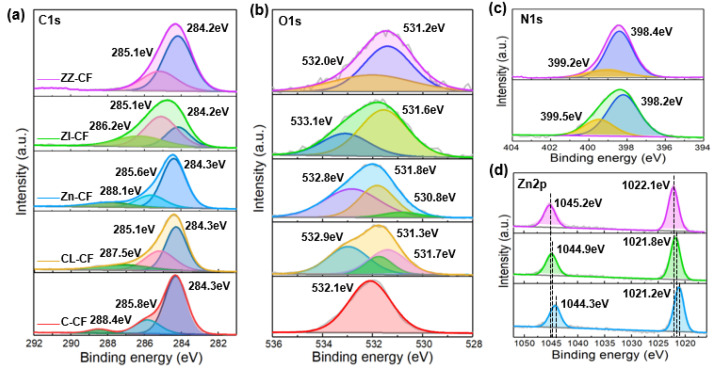
XPS spectra of (**a**) C 1s, (**b**) O 1s for C–CF, GL–CF, Zn–CF, ZI–CF, and ZZ–CF; (**c**) N 1s, and (**d**) Zn2p for Zn–CF, ZI–CF, and ZZ–CF, respectively.

To investigate the functional groups on the CF, the FTIR curves of C–CF, GL–CF, and ZZ–CF were shown in Figure 2a, respectively. Contrasting with the C–CF, it can be seen that the peaks of hydroxyl and carbonyl groups on the surface of CF modified by glucose have been observed. The characteristic peaks were obviously at 3000 cm^−1^–3600 cm^−1^, 2916 cm^−1^, and 2848 cm^−1^, which were assigned to the O–H, and C–H bonded to the surface of GL–CF [36]. The peaks attributed to the vibrational absorption of C=O and C–O were located at 1630 cm^−1^ and 1050 cm^−1^. The curve of ZZ–CF demonstrated the peaks of aromatic and aliphatic C–H stretching, associated with stretching in the imidazole rings and methyl groups at 3135 cm^−1^ and 2929 cm^−1^, respectively. The peak at 1583 cm^−1^ is assigned to C=N stretching modes whereas the entire ring stretching was relevant to the peaks at 1422 cm^−1^ and 1383 cm^−1^. The signal at 1146 cm^−1^ corresponded to the aromatic C–N stretching mode. The characteristic peaks at 994 cm^−1^ and 760 cm^−1^ are allocated to the in–plane bending mode of the imidazole ring and the aromatic bending mode of sp^2^ C–H, respectively [35]. Similarly, the peak at 684 cm^−1^ is attributed to the 2–methylimidazole ring, and the Zn–N intensity is detected at 465 cm^−1^, which was consistent with zinc ions to the linkers via nitrogen atoms [37].

The wide XPS spectrum (Figure 1d) clarified a noticeable composition variation of C–CF, GL–CF, Zn–CF, ZI–CF, and ZZ–CF. Compared to the Zn–CF, the atomic ratio of O to Zn was reduced from 3.26 to 0.71 in the ZZ–CF after coated ZIF–8 [38]. The Zn^2+^ of the ZnO surface contact with the N atoms during the ZIF–8 formation process was confirmed by the composition change of the O and N atomics in the XPS spectrum. The N content relative to Zn increased from 2.56 to 2.73%, which was connected with the seed layer of ZZ–CF. In contrast to Zn–CF and ZI–CF, the ZZ–CF XPS spectrum displayed a faint peak corresponding to the O 1s, and the atomic ratio of oxygen reduced dramatically, indicating that the ZnO seed layer was almost covered with the ZIF–8 shell. With the seed layer on the GL–CF, the content of the Zn atom was significantly increased from 5.77% to 7.76% in the ZZ–CF, contributing to the more active sites provided by the Zn^2+^ of the seed layer promoting the ZIF formation.

To further investigate the composition and electronic state of elements on the surface of C–CF, GL–CF, Zn–CF, ZI–CF, and ZZ–CF, the XPS was performed and shown in Figure 2. The spectrum of C 1s was shown in Figure 2a, which can be resolved into different individual peaks of various prepared samples. The three characteristic peaks of C–CF displayed at 284.3 eV, 285.8 eV, and 288.4 eV, corresponding to the C–C, C–O, and C=O, which were assigned to the raw carbon fibers and the residual resin of the surface of carbon fibers [39]. The binding energy of 287.5 eV, 285.1 eV, and 284.3 eV was attributed to C=O and sp^2^ carbon [40], owing to the hydrothermal reaction of CF in the glucose solution. The presence of the C–O bond corresponded to the peak at 285.6 eV for Zn–CF, whose binding energy was 0.5 eV higher than that of ZI–CF and ZZ–CF. As shown in Figure 2b, the peak at 532.1 eV of C–CF was associated with physically adsorbed O_2_ on the surface of the fiber; meanwhile, the O 1s spectra of GL–CF could be divided into three peaks at 531.3 eV, 531.7 eV, and 532.9 eV, which attributed to the O–H, O=C, and C–O corresponding to the C 1s. The peaks of Zn–CF at 530.8 eV, 531.8 eV, and 532.8 eV were assigned to the Zn–O bonds [41], Oxygen ions in the ZnO [42], and O–H, respectively. For ZI–CF, the characteristic peaks of O 1s were located at 531.6 and 533.1 eV, which were attributed to the O in the forms of Zn–OH, respectively [35]. Due to the spatial structure of ZIF on the surface of ZI–CF, oxygen was detected. Furthermore, compared to Zn–CF, peaks at 531.2 eV and 532.0 eV for ZZ–CF were assigned to the Zn–O bond and Oxygen ions [43]. The N element was exhibited in the ZI–CF and ZZ–CF, assigned to the involvement of dimethylimidazole. In the N 1s spectrum (Figure 2c), the peaks located at 398.2 eV and 399.5 eV were assigned to the C–N and Zn–N bonds of ZI–CF and ZZ–CF [42,44]. Owing to the binding energy of O–Zn–N, the shift of the N 1s peak position was obvious.

It can be seen from Figure 2d that the high–resolution XPS spectrums of Zn–CF, ZI–CF, and ZZ–CF presented two individual sharp shapes of the Zn2p peaks, which were located at 1021.2 eV and 1044.3 eV of Zn–CF assigned to Zn 2p3/2 and Zn 2p1/2, respectively [45]. The corresponding peaks of the ZI–CF curve were observed at 1021.8 eV and 1044.9 eV, whose binding energy was shifted about 0.6 eV towards higher energies, indicating the different binding energy of Zn [35]. Compared to the Zn–CF, the two characteristic peaks of ZZ–CF shifted approximately 0.9 eV, proving the heterojunction of ZZ–CF [5], which exhibited the ZIF–8 on the surface of the ZnO seed layer [35]. The higher binding energies than those of the Zn–CF and ZI–CF indicated the electronic environment around the Zn^2+^. For ZIF–8, four imidazole N atoms coordinated with a Zn^2+^, which was surrounded by O^2−^ in ZnO. The distinct ligands with the Zn^2+^ on the seed layer surface created different electronic environments, resulting in the shifted peak position [38], which was consistent with the peak shift of N 1s. Furthermore, the variation of electron density for different atoms in the as–prepared samples resulted in the shifting of peaks [37], which was consistent with the above.

**Figure 3:**
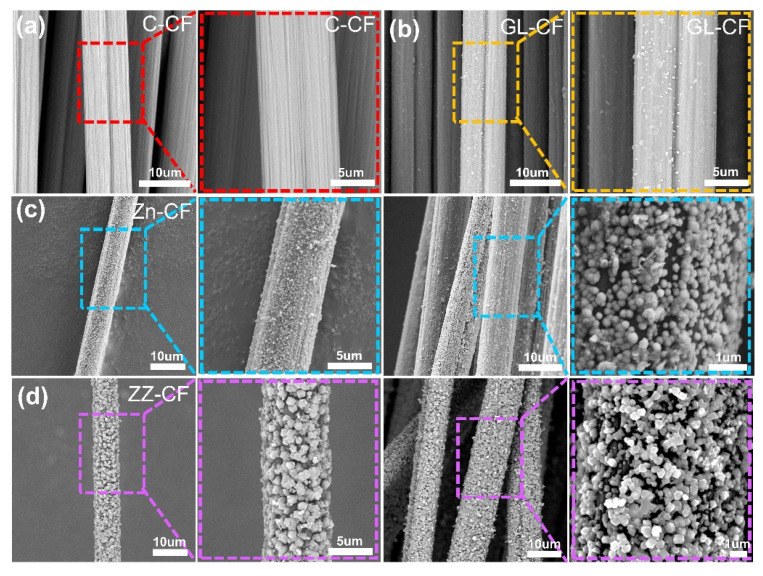
SEM images of (**a**) C–CF, (**b**) GL–CF, (**c**) Zn–CF, and (**d**) ZZ–CF, respectively.

To further investigate the distribution of the seed layer and the ZIF–8 shell structure decorated on the CF surface, SEM images were employed. Figure 3 illustrated the morphology and structure changes of different samples after treatment. As shown in Figure 3a,b, compared to the C–CF, the micelles, consisting of amphiphilic macromolecules, attached to the formation of carbon coating on the CFs surface enhancing the roughness, which provided more active sites and contributed to the in–situ growth of functional semiconductor materials. Meanwhile, the polar groups of carboxyl, carbonyl, and hydroxyl groups were produced in the process of hydrothermal carbonization, which further improved the reaction activity of CFs [46]. Furthermore, the inner seed layer was effectively cultivated on the surface of the GL–CF providing a reactive site, simultaneously. From Figure 3c, it can be observed that the growth of seed was a random distribution that contributed to the deformation of substrate guaranteeing a stable property output for photocatalyst. As shown in Figure 3d, the distinctively homogeneous growth of the ZIF shell was observed over the seed layer on the CFs fiber surface via the in–situ growth in methanol solution. The good distribution of photocatalysts without obvious agglomeration provided abundant active sites, which was consistent with the design philosophy of the composite material.

### 2.2. Photocatalytic Degradation of TC

**Figure 4:**
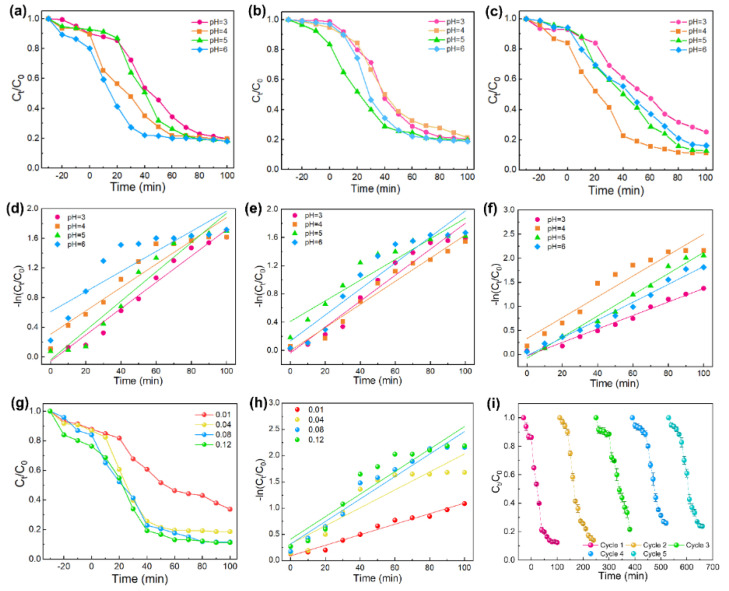
(**a**–**c**) Effect of initial solution pH; (**d**–**f**) first–order kinetic equation for Zn–CF, ZI–CF, and ZZ–CF, respectively; (**g**) Effect of photocatalyst dosage; (**h**) first–order kinetic equation with different dosage for ZZ–CF; (**i**) Cycling experiments with optimal pH and dosage of ZZ–CF.

To verify the photocatalytic performance of ZZ–CF for TC solution, the photocatalytic degradation experiments were monitored under UV–vis light irradiation after adsorption–desorption equilibrium in the dark for 30 min. In this process, 50 mL TC (50 mg/L) was adopted to determine the photocatalytic activity in the pH range of 3–6. Compared with the photodegradation efficiency of Zn–CF (82.02%) and ZI–CF (81.14%), ZZ–CF (88.47%) exhibited the highest efficiency, which was shown in Figure 4a–c. Meanwhile, the degradation kinetics of TC were evaluated by the pseudo–first–order ($-ln\frac{C_{t}}{C_{0}}=kt$), where *t* (min) is the time, *C*_0_ (mg/L) is the initial TC concentration, *Ct* (mg/L) is assigned to the concentration at *t* min, and *k* represents the rate constant of first–order kinetics, which represents the photocatalytic reaction rate [12]. As shown in Table 1, the kinetic equation of ZZ–CF proved that the photodegradation efficiency of ZZ–CF (0.0214) was the highest, compared to Zn–CF (0.0198) and ZI–CF (0.0185) at the designed range of the pH value (Figure 4d–f). This combination of the seed layer and ZIF–8 facilitated the photocatalytic performance for TC.

Furthermore, the best condition of the optimal photocatalyst was exhibited. To investigate the optimal photocatalytic conditions of the ZZ–CF photocatalyst, the mass of the photocatalyst and pH of TC solution were controlled in this reaction. Meanwhile, Figure 4a,b of contrasting experiments on Zn–CF and ZI–CF have proven that ZZ–CF displayed desired photocatalytic performance. When the photocatalyst existed, the adsorption reaction happened under the dark condition after a certain time, which could promote the photodegradation reaction. With the pH increased in the range of 3–6, similar photocatalytic efficiency can be achieved. Compared to the Zn–CF (pH = 6) and ZI–CF (pH = 6), ZZ–CF exhibited excellent photocatalytic performance at the optimal pH value of 4 (Figure 4c). However, in the photodegradation process, the excessive adsorption of TC on the photocatalyst reduced the contact between the active surface with the solution [12], resulting in a decreased photodegradation efficiency for TC. Based on the balance of prophase adsorption and subsequent photocatalytic activity, the designed ZZ–CF indicated both a superior adsorption effect and continuous excellent photocatalysis for TC, enabling photodegradation efficiency to reach 88.47%; this remarkable performance can be supported by the previous results of transient photocurrent, electrochemical impedance spectroscopy. In addition, based on the ZnO seed layer and conductive CFs, the core–shell structure of the photocatalyst reduced the transport resistance of the photoelectron and improved the photocatalytic efficiency under photoexcitation.

To obtain maximum photocatalytic activity and economics, the photocatalysts of the optimized dose were essential in water treatment [7]. The photocatalytic activity was connected with the concentration of the original solution and the dose of photocatalysts. Therefore, to understand the optimum dose, a gradient of 0.01 to 0.12 g/50 mL of ZZ–CF was added to TC solution at a concentration of 50 mg/L. It can be seen from Figure 4g that the photodegradation efficiency gradually improved with the increase in the mass of the photocatalyst, but when the mass of ZZ–CF was more than 0.08 g, the photocatalytic efficiency did not continue to improve. As shown in Figure 4h and Table 1, the TC photodegradation efficiency increased from 66.27% to 88.78% along with a photocatalyst dose from 0.01 to 0.12 g/50 mL, indicating that a plateau phase had been reached, which has a similar photodegradation rate of 0.0214 min^−1^ for 0.08 and 0.12 g/50 mL. In the reaction process, the previously increasing dose of photocatalysts could be contributed to the formation of active sites, subsequently, improving the photodegradation efficiency. While the decreased photocatalytic degradation efficiency was assigned to the excessive dose of photocatalyst, which could block the light [47]. Therefore, 0.08 g of the ZZ–CF photocatalyst was the best addition amount. Under the optimal conditions with a pH value of 4 and catalyst dosage of 0.08 g, the photocatalyst repeatability experiments were carried out for 50 mL of 50 mg/L TC. The ZZ–CF photocatalyst for the repeated experiment was collected after each photocatalysis cycle. Figure 4i showed the cycling stability of ZZ–CF, which still maintained 75.0% of the initial catalytic ability after 5 cycles.

### 2.3. Optical and Electronic Properties

**Figure 5:**
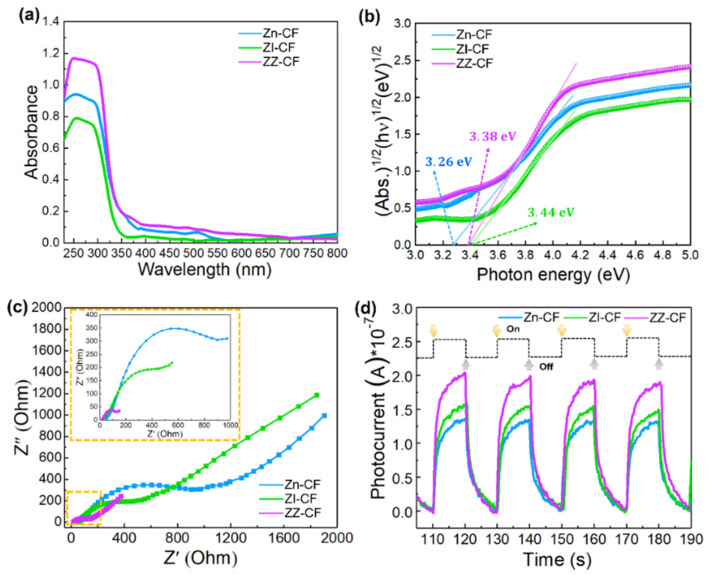
(**a**) DRS curves of Zn–CF, ZI–CF, and ZZ–CF, respectively; (**b**) Energy gap analysis of Zn–CF, ZI–CF, and ZZ–CF, respectively; (**c**) EIS and Nyquist plots with the equivalent circuit of Zn–CF, ZI–CF, and ZZ–CF, respectively; (**d**) Transient photocurrent of Zn–CF, ZI–CF, and ZZ–CF, respectively.

To demonstrate the influence of the ZIF–8 structure on photocatalytic performance, the optical property of Zn–CF, ZI–CF, and ZZ–CF were investigated by UV–vis DRS (Figure 5a). Compared with Zn–CF and ZI–CF, ZZ–CF exhibited a strong photo–absorption in the UV region with an absorption edge of around 350 nm, which was consistent with the absorption edge of Zn–CF and ZI–CF. The ragingly enhanced adsorption of UV light should result from the shell structure of the seed layer combined with ZIF–8, confirming that the extraordinary structure on the surface of the CF substrate had a positive effect on the optical properties. Meanwhile, within the matrix of the seed layer, ZnN_4_ clusters of the ZZ–CF might induce a charge transfer mechanism creating an excitonic absorption [48]. ZZ–CF displayed a similar analogous characteristic edge to Zn–CF demonstrating the successful growth of the seed layer, which approached the Zn–CF, indicating the retention of the seed layer during the formation process of the outer shell structure of ZIF–8. Furthermore, the band–gap values were evaluated by measuring the *x*–axis intercept from the linear region, which was transformed from the UV–vis absorption curves of the samples.

As shown in Figure 5b, the band gaps of Zn–CF, ZI–CF, and ZZ–CF might be 3.26 eV, 3.44 eV, and 3.38 eV, respectively. Meanwhile, both ZI–CF and ZZ–CF represented the approximate bandgap of 3.44 and 3.38 eV, exhibiting that the energy band of ZZ–CF was feebly affected by the seed layer [38]. Accordingly, compared to ZI–CF, the enhanced absorption of ZZ–CF in the UV region inspired the photocatalytic activities under UV irradiation, leading to preponderant photocatalytic efficiency [49].

The synthetic strategy has been used to fabricate a high–performance flexible photocatalyst with a core–shell based on the CFs. They benefited from active groups of the CFs and the unique structure, endowing excellent electrical conductivity of ZZ–CF. As an important evaluation parameter of photocatalyst performance, the photocatalysts were investigated on electrochemical impedance spectra (EIS) and photocurrent responses. The EIS of Zn–CF, ZI–CF, and ZZ–CF were used for clarifying the charge transfer resistance. As demonstrated in Figure 5c, the obtained Nyquist plots with smaller semicircular sizes demonstrated a lower equivalent series resistance of ZZ–CF, confirming accelerated transport and diffusion of ions and electrons. In the constructed structure of ZZ–CF, the core of CFs created conductive pathways assisting electron transfer between the photocatalysts and CF, wherein the seed layer was an active material for photochemical interactions with photoelectrons. In the meantime, the transient photocurrent curves intuitively indicated the fast response performance of different photocatalysts to light facilitating the separation and migration of electrons and holes. During the irradiation cycles, the rise and fall of the photocurrents were apparent, which was exhibited in Figure 5d. However, the photocurrent was unobvious in the dark. As soon as light irradiation was turned on, photo responses were observed owing to the sensitive transient effect of the photon–generated carrier [23]. Therefore, photocurrent curves raised slowly whilst maintaining some steady state during the light on. The photocurrent tardy declined to the original value when the light was off, indicating the continuous transfer phenomenon of charge carriers. It can be seen that, compared with the Zn–CF and ZI–CF, the photocurrent density of ZZ–CF was the strongest among all samples, indicating a stronger ability, and higher separation efficiency for the photo–generated electrons and holes [30]. The result matched with the EIS, demonstrating the superior photocatalytic properties of the ZZ–CF composite.

### 2.4. Possible Photocatalytic Degradation Mechanism

**Figure 6:**
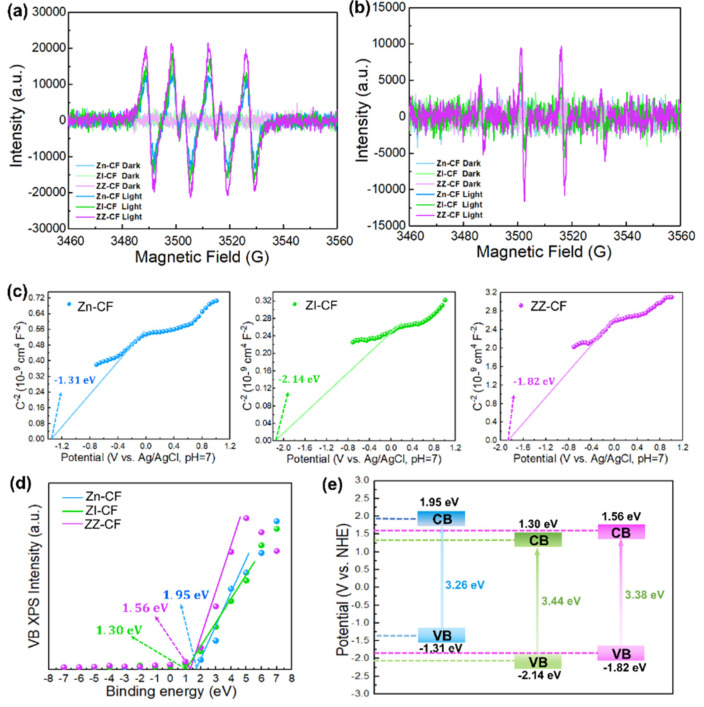
(**a**,**b**) EPR spectra of Zn–CF, ZI–CF, and ZZ–CF for ·O_2_^−^ and ·OH, respectively; (**c**) Mott–Schottky plots of Zn–CF, ZI–CF, and ZZ–CF at 1000 Hz; (**d**) VB XPS of Zn–CF, ZI–CF, and ZZ–CF; (**e**) The band alignments versus the normal hydrogen electrode (NHE) for Zn–CF, ZI–CF, and ZZ–CF.

As shown in Figure 6a,b, both ·OH and ·O_2_^−^ were generated from photocatalysts under UV irradiation, which was confirmed by the quantitative investigation of the intensity of active oxidative species, investigated by the electron spin resonance (ESR). Compared with Zn–CF and ZI–CF, ZZ–CF showed a stronger signal with characteristic peaks of ·OH and ·O_2_^−^ under irradiation. In the photocatalytic process, the production of ·O_2_^−^ was assigned to the reaction between photo–electrons and surface–adsorbed oxygen, while ·OH corresponded to the photo–holes, which could react with OH^–^. Meanwhile, the existence of ·O_2_^−^ could contribute to the conversion of ·OH. The target organic molecules could be degraded by ·OH, ·O_2_^−^ in the process of oxidation–reduction [50].

The possible mechanism for photocatalysts was proposed on the Mott–Schottky equation and VB XPS spectra. The Mott–Schottky equation was used to evaluate the conduction band of photocatalysts. It can be seen from Figure 6c that the conduction band edge potential (*E*_CB_) values of Zn–CF, ZI–CF, and ZZ–CF were inferior to the reduction potential for O_2_ to ⋅O_2_^−^ (−0.33 eV) [10]. Meanwhile, the valence band edge potential (*E*_VB_) value was calculated by the VB XPS spectra (Figure 6d), which showed the 1.95 eV, 1.30 eV, and 1.56 eV for Zn–CF, ZI–CF, and ZZ–CF, respectively. However, the potential of +2.38 eV and +2.72 eV for OH^−^ to ·OH and H_2_O to ·OH were higher than that of the photocatalysts. The band alignments versus the normal hydrogen electrode (NHE) were indicated in Figure 6e. The possible formation procedure of ·OH was attributed to the O_2_ due to the potential energy of +0.695 eV for O_2_ to H_2_O_2_, which subsequently was transformed to ·OH with the capture of electrons [51,52]. The results showed reactive species in this photocatalytic system including O_2_^−^, H+, and ·OH, which coincided with the ESR of the photocatalysts.

Based on the discussion, the photogenerated electron holes of ZZ–CF could induce a series of active free radicals to decompose TC in the solution. The possible reaction process of ZZ–CF under UV–visible light irradiation could be depicted as follows (Equations (1)–(4)):

ZZ–CF + *hv* → ZZ–CF(e^−^) + ZZ–CF(h^+^) (1)

ZZ–CF(e^−^) + O_2_ → ZZ–CF +·O_2_^−^ (2)

O_2_^−^ + H_2_O → H_2_O_2_ + ·OH (3)

O_2_^−^, H^+^, ·OH + TC → H_2_O + CO_2_ (4)

## 3. Materials and Methods

### 3.1. Materials

Acetone, ethyl alcohol, methyl alcohol, glucose, zinc acetate (Zn(CH_3_COO)_2_), ammonium hydroxide (NH_4_OH), and zinc nitrate (Zn(NO_3_)_2_) were purchased from Sinopharm Group, China, (Shanghai, China) and without further purification. 2–Methylimidazole was obtained from Aladdin Industrial Inc (Shanghai, China). Carbon fibers were purchased from Zhongfu Shenying Carbon Fiber Co., Ltd. (Lianyungang, China).

### 3.2. Synthesis of Photocatalytic Materials

The preparation process of photocatalyst (ZZ–CF) was depicted in Figure 1a. Firstly, the 10 g raw carbon fibers (R–CF) were immersed in a mixture consisting of acetone (80 mL), ethyl alcohol (80 mL), and deionized water (40 mL) for 24 h at room temperature. After that, the clean carbon fibers (C–CF) were prepared by washing three times and dried at 80 °C for 2 h. Secondly, activated carbon fibers (GL–CF) were obtained by hydrothermal reaction with the 42 g/L glucose solution. The hydrothermal reaction was carried out in a 100 mL high–temperature reactor at 220 °C for 2 h. The GL–CF was washed with distilled water and dried at 80 °C. Thirdly, the seed layer was formed in the 200 mL transparent mixture solution with 8.78 g Zn(CH_3_COO)_2_ and superfluous NH_4_OH for 24 h at room temperature, and Zn–CF was acquired after drying at 80 °C for 1 h. Subsequently, 3.275 g of Zn(NO_3_)_2_ was adequately dissolved in 120 g methyl alcohol for 30 min. Meanwhile, 7.375 g of 2–methylimidazole was dissolved in 180 mL methyl alcohol. Zn––CF was added to the 2––methylimidazole solution for 10 min, and Zn(NO_3_)_2_ solution was poured into the above mixture with stirring for 12 h at 60 °C. The ZZ––CF photocatalyst was obtained after washing and drying at 100 °C. Similarly, the ZIF–8–CF (ZI–CF) was obtained through replacing Zn–CF to GL–CF in the above synthetic process.

### 3.3. Characterization

The chemical compositions of as–prepared photocatalysts were measured by X–ray diffraction (XRD, PANalytical B.V., Amsterdam, The Netherlands) with a scan rate of 8.5 °/min from 5° to 90°, Fourier transforms infrared (FTIR, Bruker Vertex 70 instrument, Berlin, German) with a range of 400–4000 cm^−1^, and X–ray photoelectron spectroscopy (XPS, a dual anode XSAM800 spectrometer, Kyoto, Japan) with non–monochromatic Al Kα X–radiation (hυ = 1486.6 eV). Morphologies were measured by the Scanning electron micrographs (SEM, JSM–IT300, Tokyo, Japan). The UV–vis diffusion reflectance spectra (DRS, UV–3600, Shimadzu, Kyoto, Japan) were based on BaSO_4_ as reference. The concentration of the TC was tested by UV–vis spectrometer (UV, UV–3600, Shimadzu, Tokyo, Japan) with Quartz cuvette at the wavelength range of 200–500 nm. Electrochemical impedance spectroscopy (EIS) and photocurrent measurements were employed by an electrochemical workstation (CHI Instruments, CHI 760E, Shanghai, China) using a conventional three–electrode system. The free radical signals were analyzed with Electron spin resonance (ESR, Bruker A300, Billerica, MA, USA) under UV–vis light for 10 min.

## 4. Conclusions

In summary, we demonstrated the ZZ–CF enhanced photocatalytic efficiency by designing synergetic architectures linking the ZIF–8 ligand to ZnO seed layer of in–situ synthesis on carbon fiber (ZZ–CF). Based on the special structure of photocatalyst, the dissolution seed layer and release of Zn^2+^ in excessive 2–Methylimidazole methanol solution were used as the binder. Moreover, the adsorption of UV light, Schottky junction, and valence band edge potential value were affected due to the formation of ZIF–8 on the passivated surface of the seed layer, enabling the adsorption in the dark and photodegradation reached equilibrium, simultaneously. Meanwhile, the conductive and stable properties of carbon fibers exhibited the superior ability to facilitate the transfer of photogenic electrons, which could significantly enhance photocatalytic performance. Additionally, compared to the Zn–CF and ZI–CF, ZZ–CF exhibited superior photodegradation for TC with a removal efficiency of 88.47%, and demonstrated photocatalytic stability under UV–vis light irradiation. This present work demonstrated a strategy for improving the photocatalytic performances of ZZ–CF and provided useful conception into the design of the synergetic structure, which inspire further investigation on constructing hybrid photocatalyst based on reaction centers with metallic oxide of passivated surface on conductive fiber.

## Figures and Tables

**Table 1:** Comparison of different samples’ performance to various pollutants.

Sample	Mass (g)	pH	C_0_(mg/L)	V(mL)	k (min^−1^)	R^2^	Photodegradation Rate (%)
Zn–CF	0.08	3	50	50	0.0178	0.966	80.12%
Zn–CF	0.08	4	50	50	0.0158	0.903	80.13%
Zn–CF	0.08	5	50	50	0.0198	0.937	81.73%
Zn–CF	0.08	6	50	50	0.0136	0.748	82.02%
ZI–CF	0.08	3	50	50	0.0184	0.951	79.55%
ZI–CF	0.08	4	50	50	0.0164	0.964	78.60%
ZI–CF	0.08	5	50	50	0.0147	0.880	80.20%
ZI–CF	0.08	6	50	50	0.0185	0.886	81.14%
ZZ–CF	0.08	3	50	50	0.0140	0.984	74.68%
ZZ–CF	0.08	4	50	50	0.0214	0.946	88.47%
ZZ–CF	0.08	5	50	50	0.0220	0.980	87.18%
ZZ–CF	0.08	6	50	50	0.0185	0.978	83.65%
ZZ–CF	0.01	4	50	50	0.0100	0.976	66.27%
ZZ–CF	0.04	4	50	50	0.0171	0.810	81.41%
ZZ–CF	0.12	4	50	50	0.0214	0.872	88.78%

## Data Availability

Not applicable.
